# Chinese oncologists’ knowledge, attitudes and practice towards palliative care and end of life issues

**DOI:** 10.1186/s12909-016-0668-3

**Published:** 2016-05-18

**Authors:** Xiaoli Gu, Wenwu Cheng

**Affiliations:** Department of Integrated Therapy, Fudan University Shanghai Cancer Center, #270, Dong An Road, Shanghai, People’s Republic of China; Department of Oncology, Shanghai Medical College, Fudan University, Shanghai, 200032 China

**Keywords:** Oncologists’ knowledge, Palliative care, Palliative care education, Physician education

## Abstract

**Background:**

Oncologists` knowledge and attitudes to palliative care (PC) and end of life (EOF) should be highlighted in order to give them effective education. This study is intended to provide a descriptive analysis of oncologists’ knowledge, attitudes and practice toward PC and EOF issues in Mainland China.

**Methods:**

The questionnaire survey with 24 items investigating oncologists’ demographic information, knowledge and attitudes toward PC and EOF issues was conducted among Chinese Oncology clinicians.

**Results:**

The participants had a mean of 10.10 years practice in oncology. 43 (31.2 %) participants had received PC education. 73.9 % of the participants believed that PC should be considered when patients were not suitable to take surgery, radiotherapy, chemotherapy and other anti-cancer therapies. 72.5 % of the participants believed that early PC integration can improve the quality of life in patients. Most of the oncology clinicians (73.9 %) believed that the doctor-in-charge was the appropriate person to inform patients of the diagnosis. However, only 11 participants chose to inform the diagnosis and disease prognosis to the patients, whereas 39.9 % of the participants chose to disclose it to Family/Caregivers first. Besides, Chinese oncologists were obviously unfamiliar with the concepts of euthanasia and related issues.

**Conclusions:**

This study indicated the insufficient knowledge toward PC and related issues of the Chinese oncologists. More attention should be paid on the education of PC among Oncologists in Mainland China.

## Background

The growing aging population, the increasing incidence rates of cancer and the huge number of terminally ill cancer patients made palliative care (PC) important and imperative for cancer patients. In recent years, there has been an increase in PC services in developing countries, including Mainland China [1]. However, compared to Western countries, PC has not been recognized as an important specialty in Mainland China yet. PC experts have suggested that oncologists’ knowledge and attitudes should be associated with their practices in care for patients [2]. But oncologists were reported to be under the misapprehension that they have sufficiency ability to deal with patients’ symptoms. Therefore, they do not tend to refer patients to PC specialists, because PC was still misconceived as doing nothing by some medical pratictioner [3]. Acutually, onocologists’ insufficient understanding of PC service is still one of the barriers to PC development. Oncologists’ knowledge and attitudes to PC and end of life (EOF) should be highlighted in order to give them effective education.

A previous survey of experienced Chinese urban physicians showed that a significant number of these physicians did not feel competent in support care and symptom management [4]. These years witnessed the great development of PC services in China. While, there have not national population-based studies available of the knowledge and attitudes of Chinese Oncologists in PC yet. We report on a questionnaire to survey Mainland Chinese oncologists’ knowledge, attitudes practice towards PC and EOF issues.

## Methods

### Questionnaire

The study was designed as a cross-sectional, anonymous, self-administered questionnaire survey. The questionnaire was developed by researchers on the basis of literatures review [5–8]. After studying literatures, 7 PC specialists generated a total of 50 items for an item pool based on the literature reviews and discussion. To achieve validity, an expert panel (3 palliative specialists, 3 oncologists) rated the appropriateness of each item. First, experts evaluated the appropriateness of each item according to four grades. Next, the mean, minimum and maximum values were calculated, and the items with less than a mean of two and a minimum of zero were deleted. Then, the argument for the item selection was repeated including an evaluation of the way the concept was expressed. As a result, a preliminary list of 24 items was selected from the original 50 items. The final questionnaire was to assess the knowledge, attitudes and practice of the respondents covering issues related to PC. The complete questionnaire included five categories: 1) personal characteristics such as gender, age, education, practice years and etc.; 2) concept and philosophy of PC (Q1-Q9); 3) disease information disclosure and breaking bad news (Q10-Q12); 4) end of life decision making issues (Q13-Q19); 5) euthanasia and related issues (Q20- Q24). For Q6 and Q17, respondents could choose more than one items. The research team reviewed the questions for clarity. The questionnaire was written in Chinese. It took approximately 10 to 15 min for experts to complete the questionnaire. A pilot study was tested in 20 oncologists for twice to assess the reliability by using Cohen’s Kappa statistics, which was 0.85.

### Participants and procedures

In order to have a representative sample and generalizable results, the questionnaire was carried out in Sep 2014 to May 2015 in three hospitals and two national oncologic meetings held in Mainland China. Three hospitals included Fudan University Shanghai Cancer Center (FUSCC), 6^th^ people’s hospital, Shanghai Jiaotong University and Kangjian Community Health Center. FUSCC is an urban, teaching, tertiary cancer center. 6^th^ people’s hospital, Shanghai Jiaotong University is a comprehensive tertiary hospital. Survey in this hospital was conducted in Medical Oncology Department. Kangjian Community Health Center is one of the primary community hospitals. Survey in this hospital was conducted in General Medical Department. Two national oncologic meetings was the Pain Management Education meeting held in Songjiang, Shanghai and the Oncologic Nutrition Meeting held in Chongqing, Sichuan. The oncologists attending the meeting came from different hospitals all over the country in China. The inclusion criterion for the subjects was that they were registered as Chinese oncologists including PC specialists.

The questionnaire and cover letter were distributed to participants. Among 145 oncologists surveyed, 138 respondents’ answers were effective and analyzed. The overall completed response rate was 95.2 %. Participation in this study was confidential and anonymous, with consent taken after provision of a cover letter containing information details of the study. Consent to participate was indicated by the completion and return of the questionnaire. All answers were entered into a computerized database by investigator for confidentiality. Then data were analyzed by an independent investigator who was unrelated to the data collection. Only the researchers had access to the data.

This study was reviewed by Research Ethics Committee of Fudan University Shanghai Cancer Center. Since this study focused on professional staff only and has no involvement of patients, there is no requirement to get permit from ethics committee in China.

### Statistical analyses

Statistical Package for the Social Sciences software Version 16.0 for Windows (SPSS Inc., Chicago, USA) was used for statistical analysis. Descriptive statistics (proportions, means and distributions) were used as appropriate to describe participants’ characteristics and respondents. 95 % confidence intervals (95 % CI) were calculated for the mean age and mean clinical practice years in Table 1.

**Table 1:** Characteristics of participants (*N*=138)

Variables	Number	Percent	Variables	Number	Percent
Age (mean, 95 % CI)	37.30	(35.8–38.8)	Gender		
20–29	23	16.3	Male	72	52.2
30–39	62	44.0	Female	66	47.8
40–49	40	28.4	Education level		
>=50	13	9.2	Med Bachelor	54	39.1
Years in practice (mean, 95 % CI)	13.43	(11.9–15.0)	Med Master	52	37.7
0–9	57	41.3	Medical Doctor	28	20.3
10–19	41	29.7	Post-doctorate	4	2.9
>=20	40	29.7	Specialty		
Years in cancer (mean, 95 % CI)	10.10	(8.6–11.6)	Oncological surgery	7	5.1
0–9	79	57.2	Chemotherapy	65	47.1
10–19	34	24.6	Radiology	6	4.3
>=20	25	18.1	TCM^a^	9	6.5
Title			Palliative/ hospice	38	27.5
Professor	24	17.4	Others	13	9.4
Assistant professor	25	18.1	Working Region		
Attending	50	36.2	Shanghai	87	63.0
Resident	27	19.6	Non-Shanghai	51	37.0
Fellow	9	6.5	Institutions		
PC education			TCH^b^	60	43.5
Yes	43	31.2	SCH^c^	14	10.1
No	95	68.8	PCH^d^	34	24.6
			Cancer Center	30	21.7

^a^TCM, Traditional Chinese Medicine

^b^TCH, Tertiary Comprehensive Hospital;

^c^SCH, Secondary Comprehensive Hospital;

^d^PCH, Primary community hospital

## Results

### General characteristics and education background

The questionnaires of 138 respondents were analyzed. 52.2 % of them were male. 84 of the participants had post-graduate education, with an average of 13.43 years of clinical experience in oncology. The participants had been in practice for a mean of 10.10 years in oncology. 42.7 % of them had more than 10 years of oncological experience. 50 (36.2 %) participants were attending physicians, 25 (18.1 %) were assistant professors and 24 (17.4 %) were professors. 43 (31.2 %) participants had received PC education. The specialty of practice included Oncological surgery (5.1 %), Chemotherapy (47.1 %), Radiotherapy (4.3 %), Traditional Chinese Medicine (6.5 %), Palliative and hospice (27.5 %). The general characteristics details of participants were in Table 1.

### The general knowledge and attitudes on PC

Q1-Q5 was designed to describe oncologists’ general knowledge of PC. The percentage of correct answers of Q1-Q5 was from 64.5 to 93.5 %. The item with the highest percentage of correct answers (93.5 %) was Q2: PC should not be provided along with anti-cancer treatment. 55 (39.9 %) participants had the correct answer for all five questions. For Q6, respondents could select more than one item in response. Only 32.6 % of the participants agreed that referral to PC should be made when cancer was first diagnosed. 73.9 % of the participants believed that PC should be considered when patients could not undertake surgery, radiotherapy, chemotherapy and other anti-cancer therapy. 29.2 % of the participants would recommend PC for cancer patients when the patients attend the clinical first time. 72.5 % of the respondents believed that early PC integration could improve patients’ quality of life (QOL) and 54.3 % of the respondents believed that PC could improve patients’ survival. Details were shown in Table 2.

**Table 2:** General PC knowledge and attitudes

Q1-Q5 items^a^ (True, False, Unknown)			Correct		Number	Percent
Q1. Palliative care should be provided for patients for whom no curative treatments are available						
Q2. Palliative care should not be provided alongside anti-cancer treatment			F		129	93.5
Q3. Different people have different ideas about PC			T		124	89.9
Q4. Patients who receive PC must accept death			F		89	64.5
Q5. Palliative care is the same as hospice care			F		117	84.8
Participants who answered everything correctly					55	39.9
Q6.^b^ In your opinion, under what conditions should cancer patients receive PC?						
A. When cancer is first diagnosed			–		45	32.6
B.When patients can no longer undergo surgery, radiotherapy, chemotherapy and other anti-cancer therapy			–		102	73.9
C. When patients’ symptoms can no longer be controlled			–		77	55.8
D. When patients are mentally disabled			–		65	47.1
E. When patients proactively request PC			–		57	41.3
F. When the estimated survival length is less than 3 months			–		80	58.0
G. When the estimated survival length is less than 6 months			–		48	34.8
H. Others			–		13	9.4
Q7-Q9 items^c^	Yes		No		Depends	
	N	%	N	%	N	%
Q7.Would you recommend PC for cancer patients who attend clinic for the first time?	40	29.2	15	11.7	81	59.1
Q8.Do you believe that PC can improve patients’ survival?	75	54.3	8	5.8	55	39.3
Q9.Do you believe that early PC integration can improve patients’ QOL?	100	72.5	9	6.5	29	21.0

^a^Q1-Q5, Respondents could respond with True, False, or Unknown. “N” represents the number of respondents who answered correctly

^b^Q6, Respondents could select more than one item in response. Thus, the percentages of Q6 add up to more than 100 %

^c^Q7-Q9, Respondents could select only one answer

### Disease information disclosure preference

The third part of the questionnaire dealt with questions aiming to identify participants’ attitudes and practice toward disease information disclosure and breaking bad news (Q10-Q12). 44.2 % participants decided to inform patients’ unfavorable prognosis according to the specific situations. Although, most of the oncology clinicians (73.9 %) believed that the doctor-in-charge was the appropriate one to inform the patient of the diagnosis. Only 11 participants chose to inform the diagnosis and prognosis to the patients first, whereas 39.9 % of the participants chose to disclose it to Family/Caregivers first. Details were shown in Table 3.

**Table 3:** Disease information disclosure and breaking bad news

Q10-Q12 items^a^	Number	Percent
Q10. Do you believe that you should inform patients of an unfavorable prognosis?		
Yes	26	18.8
No	2	1.4
It depends on patients’ wishes	29	21.0
It depends on family/caregivers’ wishes	20	14.5
It depends on situations^b^	61	44.2
Q11. Who should disclose information to patients?		
Doctor in charge	102	73.9
Family/Caregivers	30	21.7
Social volunteers	3	2.2
Others	3	2.2
Q12. Which person would you prefer to inform about the diagnosis and prognosis?		
Patients	11	8.0
Family/Caregivers	55	39.9
It depends on patients’ wishes	34	24.6
It depends on family/caregivers wishes	38	27.5

^a^Q10-Q12,Respondents could select only one answer

^b^It depends on the situation meant to decide on a case-by-case basis by considering the physical and psychological conditions, religion, and the cultural background of each individual

### Decision making and end-of-life issues

Q13-Q24 was to describe participants’ knowledge and attitudes on decision making and EOF issues. More than 50 % of the participants had no idea of advanced directives (ADs) and do not resuscitate order (DNR). Only 7.2 % of the participants approved that terminally ill cancer patients should received Cardiopulmonary Resuscitation (CPR) at the terminal stage. 58.7 % of the participants though it was appropriate to discuss case-by-case according to the situations. 52.9 % of them would approve patients’ wish when patients and family had conflicts on the decision making. If the patient was no longer competent and the family’s wishes conflicted with those previous expressed by the patient, 38.4 % of the participants would choose to support patients’ wish. Details were in shown in Table 4. Only 20 (14.5 %) participants were reported to be familiarity with all five concepts listed in the Table 5. The greatest proportion (77.5 %) reported being familiar with euthanasia. Physician-assisted suicide was the most unfamiliar concept, known by only 34 participants (24.6 %).

**Table 4:** Decision making and end of life issues

Q13-Q15 items	Yes		No		Not clearly	
	N	%	N	%	N	%
Q13.Do you know what advanced directives (ADs) is?	37	26.8	69	50.0	32	23.2
Q14.Do you know what a do not resuscitate (DNR) order is?	26	18.8	80	58.0	32	23.2
Q15.Do you think you should follow a patient’s wish when he prefers to forgo life sustaining treatments?	75	54.3	9	6.5	54	39.1
Q16-Q17 items					N	%
Q16. Do you approve using CPR for terminally ill cancer patients?						
Yes					10	7.2
No					47	34.1
It depends on the situation^b^					81	58.7
Q17. ^a^What factors do you believe will affect a patient and family’s decision?						
A. Disease prognosis					99	71.7
B. Symptom burden					58	42.0
C. Other disease and comorbidities					67	48.6
D. Religious beliefs					75	54.3
E. Economic status					88	63.8
F. The patient’s own wishes/preferences					82	59.4
G. Past experiences with death					69	50.0
All selected					32	23.2
Q18-Q19						
Q18. Should a conflict arise between the patient’s wishes and the family’s wishes in the decision-making process, who would you support?						
Patients					73	52.9
Family					26	18.8
It depends on the situation^b^					39	28.3
Q19. If the patient is no longer competent and the family’s wishes conflict with those previous expressed by the patient, who would you support?						
Patients					53	38.4
Family					40	29.0
It depends on situation					45	32.6

^a^Q17: Respondents could select more than one item in response. Thus, the percentages of Q6 add up to more than 100 % .^b^It depends on the situation meant to decide on a case-by-case basis by considering the physical and psychological conditions, religion, and the cultural background of each individual

**Table 5:** Euthanasia and related issues

Q 20–24	Yes		No		Not clearly	
	N	%	N	%	N	%
Q20.Do you know what euthanasia is?	107	77.5	4	2.9	27	19.6
Q21.Do you know what active euthanasia is?	63	45.7	15	10.9	60	43.5
Q22.Do you know what passive euthanasia is?	44	31.9	23	16.7	71	51.4
Q23.Do you know what physician-assisted suicide is?	34	24.6	29	20.6	75	54.3
Q24.Do you know what palliative sedation is?	44	31.9	27	19.6	47.5	48.6
Participants familiar with all concepts	20	14.5				

## Discussion

This is the first study to assess Chinese oncologists’ knowledge, attitudes and practice towards PC and EOF issues. Our findings were some of the supplement of the previous studies conducted in Asian countries on physicians’ attitudes.

Participants in this study had insufficient knowledge about PC. The insufficiency knowledge on PC may due to the lacking of formal PC education and training. Most worldwide physicians agree that current undergraduate and postgraduate programs do not provide sufficient education on PC [9]. In comparison to Western countries, PC education had not yet been institutionalized with respect to either the medical educational system or gaining the official status that other medical specialties hold, even oncology. PC has not been recognized as an important specialty in China yet. Although, more than 80 % of Chinese interns in previous research felt that more education about PC should be included in the basic medical curriculum and clinical intern training [10]. In this survey, a large number of participants (68.8 %) had never received any formal education about PC. The lack of knowledge of PC among oncologists is one of the most common barriers to high quality PC services. More attention urgently needs to be paid on PC knowledge among the Oncologists in Mainland China.

Although a number of publications have recommended early access to PC for cancer patients. PC was reported to be implemented late in the disease trajectory in previous studies [11]. The timing of referral to PC is a complex and dynamic process involving a wide range of dimensions [12]. Theoretically, oncologists’ attitudes toward PC is one of the factors contributing to referrals time [13, 14]. In our survey, only 29.2 % of the participants would recommend PC for cancer patients when the patients attended the clinical first time. Although 72.5 % believed that early PC integration could improve patients’ QOL. This phenomenon indicated that further education regarding early PC integration should focus not only on patients but also on their doctor-in-charge.

Disease information disclosure, especially how to breaking bad news is one of the important issues in PC*.* According to the tort liability laws of China (Chapter VII liability for damages caused by medical treatment, Number 55), the medical staff shall explain to patients the situation regarding his illness and the measures for medical treatment. If it was not advisable to explain directly to patients, the explanation of the same should be served to their close family members which written consent should be obtained. Most physicians in Northern Europe and the US would usually reveal the diagnosis to the cancer patient. The way to deliver bad news in oncology is influenced by legal, ethical and cultural aspects [15]. A previous study on 60 oncology clinicians shown that only 40.5 % of oncologists believed patients with terminal illness should be informed of the truth [4, 16]. The situation was similar, in our survey, although most of the oncology clinicians (73.9 %) believed that the doctor-in-charge was the appropriate one to inform the patient of the diagnosis, Only 11 (8.0 %) clinicians believed that patients should be the first choice for information disclosure. Oncologists often found themselves in conflict with their medical teaching, cultural values, patient desires, family demands, or spiritual beliefs. For Chinese oncologists, disclosing diagnosis and prognosis to patients represented a big challenge, because they were confronted with a family-centered model of decision making [17]. Chinese oncologists had to face the dilemma of respecting patients and families’ concerns about beneficence [2]. Whether family’s points of view have some coincident with patients’ standpoint need to be conducted by further researches focused on patients’ attitudes.

ADs serves as a legal document that allows competent patients to give instructions regarding the health care they would like to receive during a time crisis when they will not be competent to make their own decisions. Although ADs have been widely advocated in Western countries, such as the USA, Canada, UK, the Netherlands, and Switzerland, only a few studies dealing with ADs in Asian countries have been reported [18–21]. Most of these studies focused on the attitudes of patients towards Ads. Thus, little is known concerning the attitudes of Asian physicians, especially in Mainland China, where there is no legalization of ADs [22, 23]. According to our survey, Chinese oncologists had insufficient knowledge about AD policy, much less knowledge than other Asian studies. But the ration was similar to a previous survey focused on medical staff’s attitudes of ADs in Mainland China (26.8 % vs 16.7 %). There exists a large gap in the degree of familiarity with ADs between Western and Eastern countries. The large gap also exists between Mainland China and other Asian countries.

The last part of the questionnaire gauges physicians’ familiarity with five end-of-life issues: euthanasia, active euthanasia, passive euthanasia, physician-assisted suicide, and palliative sedation. Chinese oncologists were largely unfamiliar with most of the concepts presented. As for questions such as oncologists’ attitudes and their intention to practice euthanasia were not included in the survey, because euthanasia and related end of life issues remains sensitive and controversial issue in Mainland China.

### Limitations

The study had some limitations. First, the sample size of this survey was relatively small. The opinions expressed in this survey could not represent those of all Chinese oncologists. Second, the survey only evaluated oncologists’ general knowledge and attitudes. This was due both to the short length of the survey and also to the intentionally superficial nature of the questions included. The questions did not delve deeply into how participants might think of certain concepts for two reasons. First, certain topics for example ADs, was still controversial and it was difficult to discuss the topic in detail in Mainland China. Second, many Chinese doctors may not have extensive knowledge of what ADs is, and asking more detailed questions without first establishing a clear definition would not be productive. To provide more information on specific issues, further education and surveys should be developed, and relevant factors should be analyzed for these issues.

## Conclusions

This study provided a descriptive analysis of oncologists’ knowledge, attitudes and practice toward PC and EOF issues in Mainland China. Chinese oncologists’ knowledge toward PC and related issues were insufficient. To provide more information on specific issues, further surveys should be developed, and relevant factors should be analyzed for these issues. And further education program including knowledge and practical recommendations on PC urgently needs more attention in Mainland China.

## Abbreviation

PC: palliative care
EOF: end of life
Q1-Q24: Question 1-Question 24
FUSCC: Fudan University Shanghai Cancer Center
QOL: quality of life
ADs: Advanced directives
DNR: do not resuscitate order
TCM: Traditional Chinese Medicine
TCH: Tertiary Comprehensive Hospital
SCH: Secondary Comprehensive Hospital
PCH: Primary community hospital
